# Over the counter CBD products in Germany: an exploratory survey about consumption patterns and health related effects

**DOI:** 10.3389/fphar.2025.1571025

**Published:** 2025-05-21

**Authors:** Anaelle Weissgerber, Maren Hermanns-Clausen, Evelyn Lamy

**Affiliations:** ^1^ Molecular Preventive Medicine, University Medical Center and Faculty of Medicine – University of Freiburg, Freiburg, Germany; ^2^ Poisons Information Center, Department of General Pediatrics, Adolescent Medicine and Neonatology, University Medical Center and Faculty of Medicine – University of Freiburg, Freiburg, Germany; ^3^ Professorship for Research of Environment-related Mechanisms of Action on Health (Exposome Research), Faculty of Medicine, University of Augsburg, Augsburg, Germany

**Keywords:** cannabidiol, cbd, Germany, over-the-counter, online survey, cannabis, wellbeing, general health

## Abstract

Over-the-counter cannabidiol (CBD) products are showing substantial growth in marked share in recent years. However, the knowledge about health effects of these products is currently weak. In an explorative, retrospective online survey, the reasons for consumption, possible health-promoting or therapeutic effects, and adverse effects among CBD consumers in Germany were investigated. The anonymous questionnaire was accessible from 21 February 2023 to 20 June 2023. Participants (n = 208) were recruited mainly via social internet platforms. The study data was collected and managed using SoSci-Survey^®^. The study group was predominantly female (66%), between 41–60 years old (46%), and most of them were chronic CBD consumers (>3 months, at least daily). The principal mode of CBD use was oil (36%) with a CBD concentration between 10%–20%. Overall, the CBD products were mostly reported to have a positive effect on health. One of the main reasons for use was for improving physical and mental capacities. Here, the effect of CBD was rated effective for helping the general state of health (89%), and for the wellbeing (89%). In 79% of cases, the CBD product was rated as effective in alleviating disease symptoms. Improvements were reported especially in relief from pain (general, chronic, muscle and joint pain), or psychological symptoms (sleep disorders, nervousness, anxiety). Reported adverse effects were mainly dry mouth and sleepiness, but for 69% of the participants, no adverse effects were reported. A strong belief in the efficacy of dietary supplements, natural remedies, and CBD products among users suggests that expectation and placebo effects may have played a relevant role in the reported outcomes. This should be considered when interpreting the results and underlines the need for further controlled studies to differentiate pharmacological effects from psychological influences.

## Introduction

### Background

More than 113 active cannabinoids have been identified from the *Cannabis sativa* plant. Cannabidiol (CBD) and delta 9-tetrahydrocannabinol (Δ9-THC) appear to be the main bioactive agents (Cather and Cather, 2020). While THC has been known for centuries for its psychoactive effects, CBD has recently become the focus of medical research (Crocq, 2020). It has no known psychoactive effects and thus no relevant potential for abuse. However, small pilot studies and exploratory studies suggest that CBD may affect pain perception (Porter et al., 2021), improve appetite, sleep and memory, or help with anxiety (Shannon et al., 2019). In a mouse model, CBD showed the potential to improve immune function (Burstein, 2015). There is also some discussion on its potential usefulness in the treatment of epilepsy (Arzimanoglou et al., 2020) or Alzheimer’s disease (Watt and Karl, 2017). A protective or therapeutic effect in cancer is also currently being researched (Heider et al., 2022).

In Germany, there is only one approved pharmaceutical product, Epidiolex, which is used up to a dose of 20 mg/kg/day to treat two forms of childhood epilepsy (Dravet-syndrome and Lennox-Gastaut syndrome), and up to 25 mg/kg/day to treat tuberous sclerosis complex (TSC)-associated seizures (Rote Liste Service GmbH, 2024).

CBD is also available as over-the-counter (OTC) products in the form of aroma oil, capsules, chewing gums, drinks, cosmetics, tobacco, or flowers. These products are mainly sold on the internet with a 5%–40% CBD content (Engeli et al., 2025). The current legal situation in Germany of these OTC products is complex. In principle, the CBD products are legal to sell if they contain less than 0.3% THC. The sale of CBD products in processed forms such as oil, cosmetics, liquid and capsules is allowed, while unprocessed CBD products such as flowers, cigarettes and tea are not (Federal Court of justice (2022). It is important to note that, according to the Federal Institute for Risk Assessment, the sale of CBD in terms of foods or food supplements is not allowed in Germany, since consumption is currently classified as unsafe (Bundesinstitut für Risikobewertung, 2022). Due to existing data gaps on the health effects, the assessment as a novel food has also been suspended, according to the Novel food catalogue of the European Commission (2025).

Limited study evidence suggests that CBD is well tolerated and associated with relatively few adverse effects, but cases of elevated liver enzymes, decreased appetite, diarrhea, somnolence and sedation have been reported (Chesney et al., 2020).

Based on our literature search, studies on the use of CBD products among the general population have been conducted across the following countries: Switzerland (Zobel and Marthaler, 2017), France (Fortin et al., 2021), United Kingdom (Moltke and Hindocha, 2021), and United States (Corroon and Phillips, 2018). These studies highlighted the fact that many consumers appear to be using CBD to improve their wellbeing, or for symptoms such as pain, stress, and anxiety. CBD products have also been used as a self-medication for a wide range of diseases. A recent survey in Germany found that over 11% of the population had used CBD-containing products, primarily for stress and pain relief, while the majority perceived these products as health-promoting and associated with low risk (Geppert et al., 2023).

### Objective

This exploratory, retrospective online survey aimed at collecting new data on OTC CBD products among CBD users in Germany. Specifically, we aimed to understand the reasons for consumption and get more detailed insight into potential health-promoting, symptom-relieving, and adverse effects of these products. We hypothesized that health-related reasons would be one of the primary drivers for CBD consumption, but that CBD use would also frequently occur in the context of managing a medical condition.

## Methods

An open online survey was developed and approved by the Ethics Committee of the University of Freiburg (ethical vote No. 23–1008-S1). The study data was collected and managed using Sosci Survey^®^.

Participants were recruited through online advertisements, flyers distributed at the Albert Ludwig University of Freiburg and the University Medical Center Freiburg, CBD user forums, self-help groups, and social media platforms like Facebook^®^. Some participants were also recruited via CBD retailers. Interested individuals received a QR code or link with a brief description of the study to access the anonymous questionnaire. Posting the survey link in various Facebook^®^ groups related to CBD, health conditions, and alternative therapies proved to be the most effective recruitment strategy.

The anonymous questionnaire was available from 21 February 2023, to 20 June 2023. It was a voluntary survey, with no incentives offered. The exploratory survey consisted of 10 pages and a total of 45 questions. The questionnaire started outlining the study’s objectives and inclusion criteria. It included a mix of yes/no questions and multiple-choice items, with some allowing respondents to provide alternative answers if none of the given options applied. The questions covered demographics, CBD consumption patterns, associations with cannabis use, and participants’ knowledge of CBD. Participants were also asked about their reasons for consumption, perceived health benefits or therapeutic effects, impact on quality of life, and potential adverse effects. All questions were optional, allowing participants to skip a question if they preferred not to answer.

Due to the exploratory nature of the study, a formal power calculation was not conducted. Instead, the minimum number of participants was set at 200. The inclusion criteria for the study were being at least 18 years old, identifying as male or female, and being a consumer of OTC CBD products. The exclusion criteria included individuals who were unable to provide informed consent, and those who did not reside in Germany. Descriptive statistics were used to describe the collected data.

## Results

A total of 293 questionnaires were returned. The completion rate was about 139/293. Some of the respondents did not respect the inclusion criteria and were thus excluded from evaluation: being able to give informed consent, and being over 18 years of age (n = 4), being male or female (n = 4), living in Germany (n = 4), having used CBD before (n = 13), using dronabinol instead of an OTC CBD product (n = 1), and answering the questionnaire on behalf of a child (n = 1). 56 other respondents confirmed an age over 18 years and their ability to give consent, but did not provide further information. After reviewing the inclusion and exclusion criteria, 208 participants were included in the final study population.

### Demographics

The respondents (Table 1) were predominantly female (138; 66%), most of them lived in the state Baden-Württemberg (74; 36%), and were aged between 41 and 60 years old (96; 46%). Most were employed (114; 55%) and had completed vocational training (47; 23%).

**TABLE 1:** Sociodemographic characteristics of the survey respondents (n = 208).

Demographic characteristics	n (%)
Gender	
Male	70 (34)
Female	138 (66)
Age (years)	
18–25	26 (13)
26–40	52 (25)
41–60	95 (46)
61–67	24 (12)
>67	11 (5)
Location in Germany	
Baden-Württemberg	74 (36)
Bayern	25 (12)
Berlin	11 (5)
Niedersachsen	15 (7)
Nordrhein-Westfalen	34 (16)
Rheinland-Pfalz	11 (5)
Others	38 (18)
Employment status	
In apprenticeship	3 (1)
Student	25 (12)
Employed	114 (55)
Unemployed, looking for work	6 (3)
Unemployed, not looking for work	21 (10)
Retired	39 (19)
Education	
Secondary modern school certificate	16 (8)
Secondary school certificate	33 (16)
Technical college certificate	19 (9)
A-levels	31 (15)
Completed vocational training	47 (23)
Diploma	20 (10)
Bachelor	21(10)
Master	10 (5)
Magister	3 (1)
Doctorate	1 (0)
Not answered	7 (3)

### CBD use pattern

**FIGURE 1:**
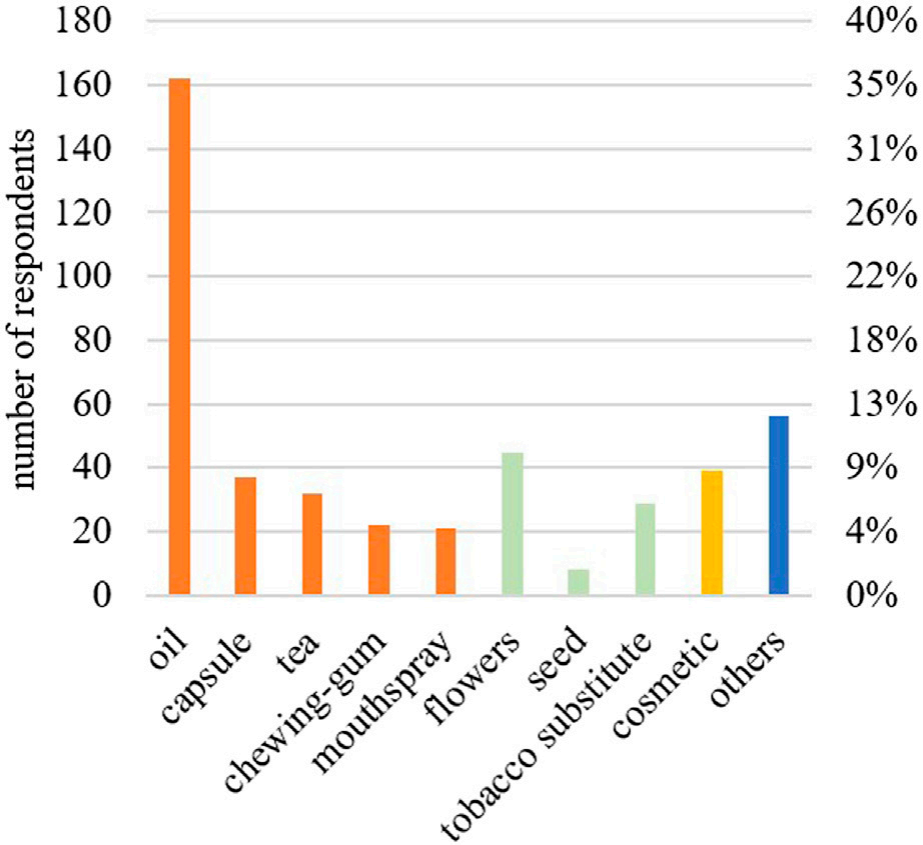
Forms of over-the-counter CBD products used by the respondents (n = 451). Oral forms are given in orange, inhaled forms in green, dermal forms in yellow, and others in blue.

A total of 54% of the respondents had experimented with more than one form of CBD consumption. The main form of CBD use was oil (162; 36%), followed by flowers (45; 10%), and cosmetics (39; 9%) (Figure 1). Oral intake was the preferred form of consumption, followed by inhalation. The concentration of CBD according to the labelling on the product (Table 2) was mainly (73; 50%) reported as 10%–20% CBD. Subjects who preferred CBD oil mentioned its ease of use, its mild taste, and the perception that it is a healthier and cleaner option compared to other forms of CBD consumption. On the other hand, those who preferred CBD flowers appreciated the stronger taste, and considered it as an alternative to traditional cannabis or tobacco use. The most popular CBD purchase locations were the internet (103; 72%), and specialized shops (26; 18%). In terms of money spent, 94 participants (68%) spent less than 50 euros on their CBD purchases per month. A high proportion of users (116; 78%) could be classified as chronic consumers, having used CBD for over 3 months. In total, 72 respondents (50%) took CBD at least once a day, 40 (28%) took it as needed. A total of 107 participants (73%) took their CBD products independently of meals.

**TABLE 2:** Pattern of CBD consumption by the survey respondents.

Pattern of CBD consumption	n (%)
CBD purchase locations (n = 143)	
Shop (in the country)	22 (15)
Shop (outside the country)	4 (3)
Internet (in the country)	79 (55)
Internet (outside the country)	24 (17)
Drugstore	6 (4)
Pharmacy	8 (6)
CBD amount (n = 146)	
<10% CBD	48 (33)
10%–20% CBD	73 (50)
>20% CBD	10 (7)
Others	15 (10)
Money spent per month (n = 138)	
<50 euros	94 (68)
50–100 euros	34 (25)
>100 euros	10 (7)
Duration of use (n = 149)	
<3 months	33 (22)
>3 months	116 (78)
Regularity of intake (n = 145)	
Irregular	16 (11)
As needed	40 (28)
≥1x daily	72 (50)
Between 1x a week and < daily	15 (10)
Others	2 (1)
Relation to meal (n = 147)	
Independent from the meal	107 (73)
>2 h apart from the meal	7 (5)
1–2 h before the meal	27 (18)
During the meal	6 (4)

### Personal view, factual knowledge, and safety of the CBD products

Overall, 81% of the respondents (113) reported a strong belief in the positive effects of CBD products. They were also more likely to use herbal remedies than synthetic chemical drugs (101; 73%). While many respondents (90; 65%) were concerned about the current state of information available about CBD, 102 respondents (74%) expressed their wish for more information. Despite these concerns, 128 (92%) of the respondents wanted CBD products to remain available as OTC products to the public; 89 (64%) were without doubts about the product’s safety. The respondents were most often introduced to CBD by friends or family (n = 65) or the internet (n = 65).

**FIGURE 2:**
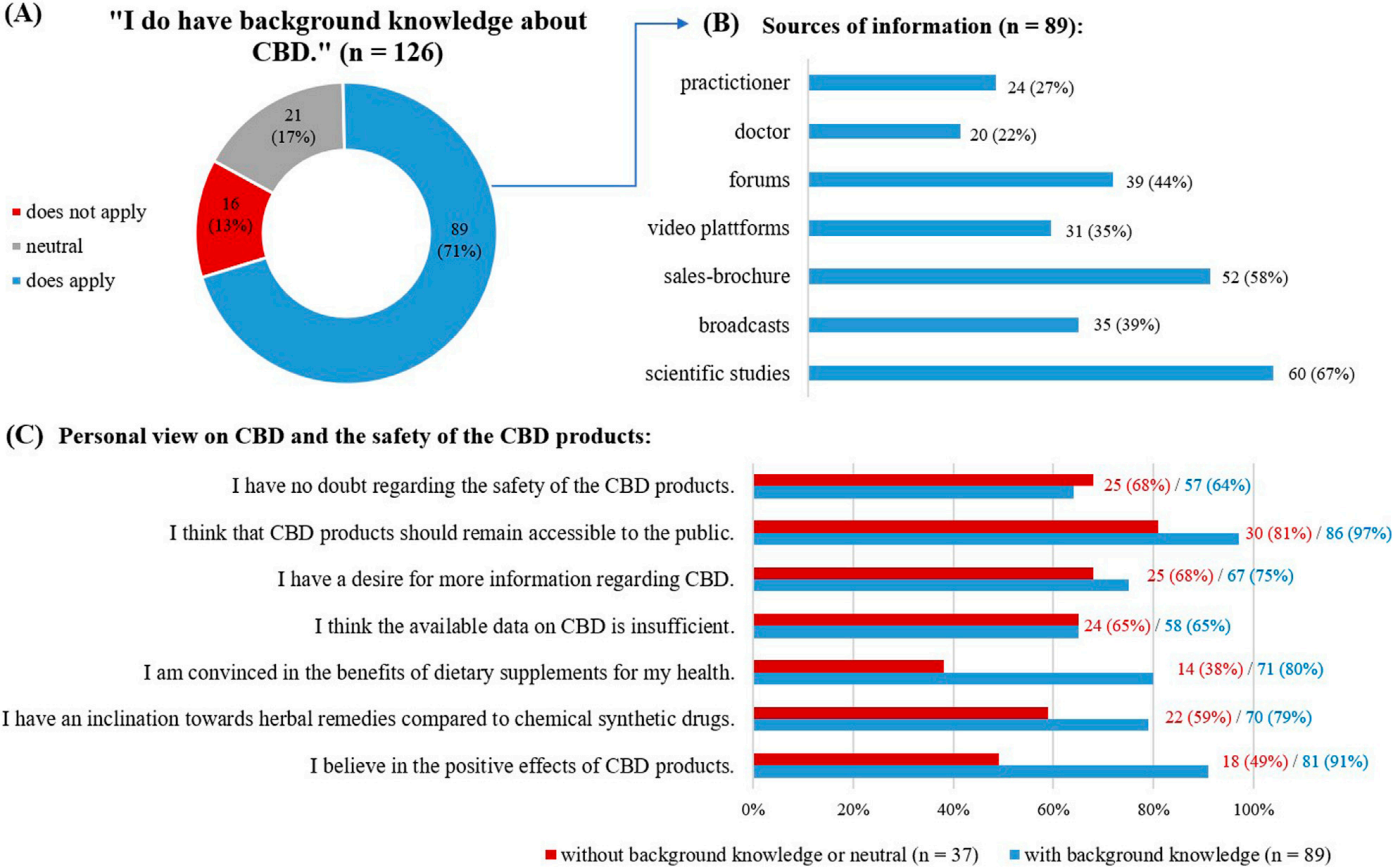
Answers given concerning background knowledge on CBD. **(A)** Self evaluation on background knowledge about CBD, as given in absolute numbers and in percentage. **(B)** Sources of information used by the respondents with background knowledge, multiple choice possible. **(C)** View on CBD and the safety of the CBD products.

Most respondents (89; 71%) reported having some background knowledge on CBD products (Figure 2A) with varied information sources (Figure 2B), including scientific studies (60; 67%), internet forums (39; 44%) and sales brochures (52; 58%).

Comparing the responses of those with self-reported background knowledge on CBD to those without (Figure 2C), the first group was more likely to have a strong belief in the positive effects of CBD (81; 91% versus 18; 49%), to use herbal remedies than synthetic chemical drugs (70; 79% versus 22; 59%), and to believe in the benefits of dietary supplements (71; 80% versus 14; 38%).

### Overall subjective efficacy of the CBD product

**FIGURE 3:**
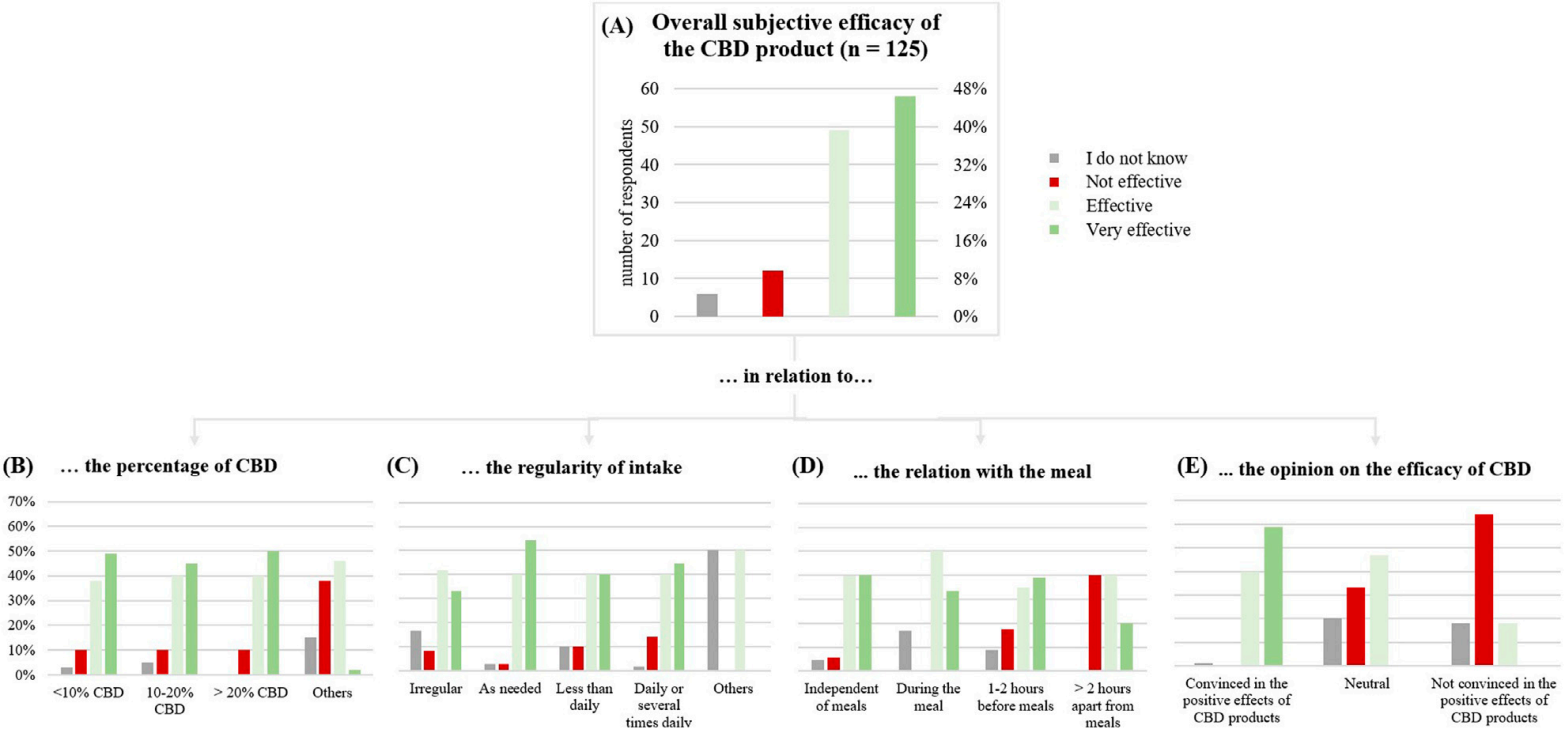
**(A)** Subjective general effect of the CBD product on the test subjects (n = 125). **(B)** in relation to the percentage of CBD. **(C)** in relation to the regularity of intake. **(D)** in relation with the meal. **(E)** in relation to the opinion on the efficacy of CBD.

Overall, 107 respondents (85%) rated the CBD products as effective (Figure 3). The effect of the CBD product was reported to set in quickly for 44 (38%) of users (after the first application).

Products with less than 10% CBD were rated as effective by 87% (5% ineffective); for products with 10%–20% CBD, 85% reported efficacy (10% ineffective). Products containing more than 20% CBD, were rated effective of 90% of users (10% ineffective).

Taking CBD as needed appeared to be more effective than irregular use (75% versus 94%) and daily use was rated more effective than less frequent use (80% versus 85%).

Respondents convinced of CBD’s positive effects rated it as effective in 99% of cases, while only 18% of those unconvinced agreed. Notably, consumers taking CBD for symptom treatment were the main group convinced of the positive effects.

### Perceived CBD efficacy for improving physical and mental capacities

Respondents often acknowledged multiple reasons for consuming CBD. In our survey, it was possible to indicate multiple reasons for consumption.

**FIGURE 4:**
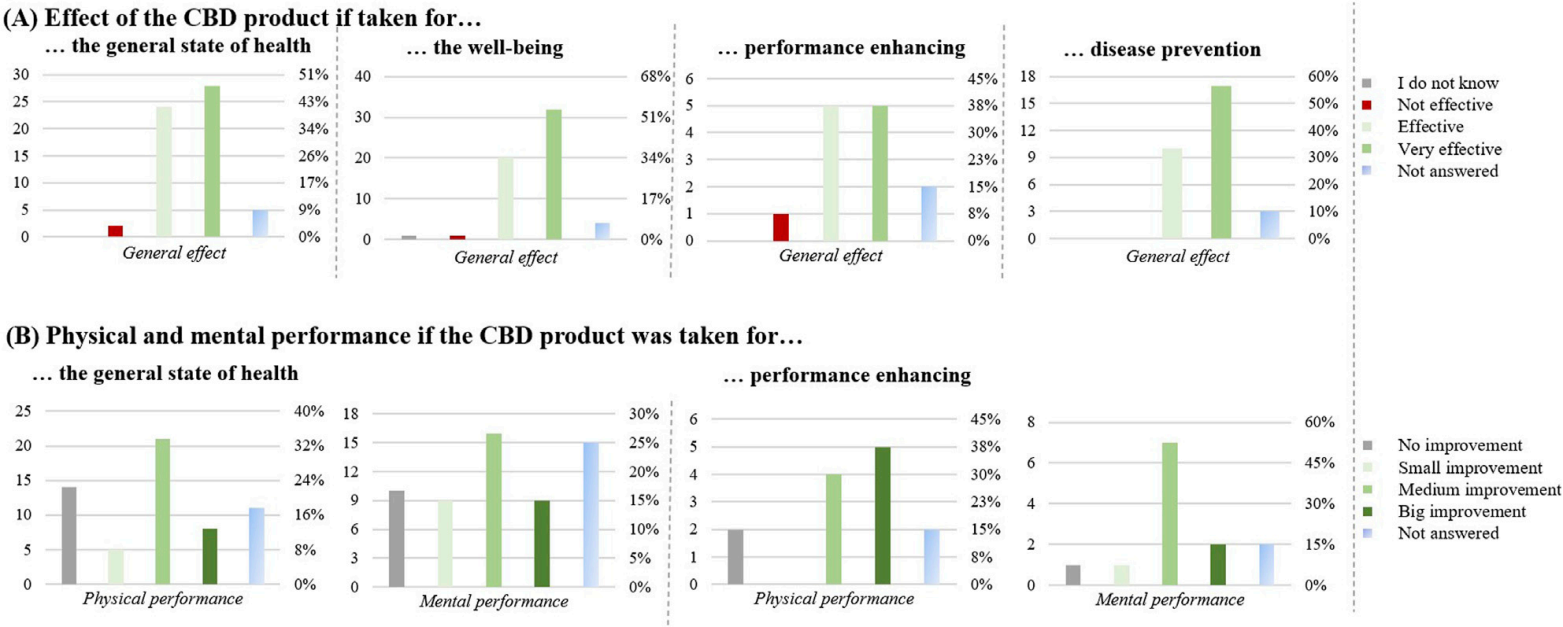
Effect of the CBD products for improving physical and mental capacities (n = 86). **(A)** Effect of the CBD product if taken for: the general state of health (n = 59), the well-being (n = 58), performance enhancing (n = 13) and disease prevention (n = 30). **(B)** Physical and mental performance if the CBD product was taken for: the general state of health (n = 59) and performance enhancing (n = 13). The graphs show the results in number of respondents (left) and in percentage (right).

One of the main reasons (Figure 4) was for improving physical and mental capacities (n = 86): for the general state of health (n = 59), for the wellbeing (n = 58), for performance enhancing (n = 13), and for disease prevention (n = 30).

For general health and wellbeing, the CBD product was perceived to be effective in most cases (52; 88%, and 52; 89%, respectively). Compared to that, with 10 respondents (77%) the CBD product was perceived less effective for performance enhancement.

For the general state of health, 40 respondents (67%) also noticed an improvement in their physical condition. 21 respondents (36%) reported a medium improvement in performance enhancement of the body and 16 respondents (27%) reported a medium improvement in mental performance enhancement. For the wellbeing, 47 respondents (90%) noticed an improvement in their wellbeing. Regarding performance enhancement, 9 respondents (69%) found a medium/big improvement in physical performance and 7 (54%) a medium/big improvement in mental performance. Finally, 17 respondents (90%) found the product effective for disease prevention.

### Perceived CBD efficacy for improving health-related issues

**FIGURE 5:**
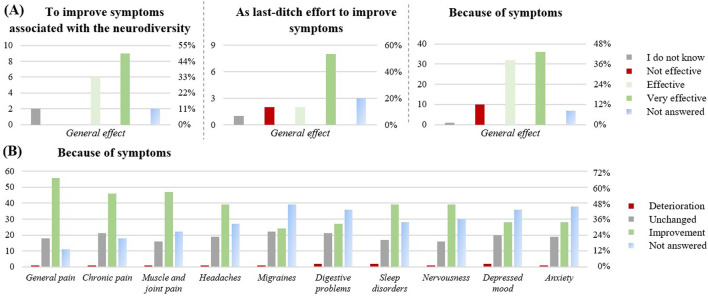
Effect of the CBD products on health-related issues (n = 100). **(A)** effect of the CBD product in relation to the different health-related reasons to take CBD: to improve symptoms associated with the neurodiversity like autism or ADHD (n = 19), as last-ditch effort to improve symptoms (n =16), Because of symptoms (n = 86). **(B)** Perceived symptom relief upon CBD intake, when CBD was taken because of symptoms (n = 86). The graphs show the results in number of respondents (left) and in percentage (right).

Another motivation for using CBD (Figure 5) was for health-related issues (n = 100): to improve symptoms associated with neurodiversity such as autism or attention deficit hyperactivity disorder (ADHD; n = 19), as a last-ditch effort to improve disease symptoms (n = 16), to support a prescribed medicine (n = 6) or because of disease symptoms (n = 86).

For the improvement of symptoms associated with neurodiversity (Figure 5A), 15 respondents (79%) found the product effective, an improvement of the ability to stay focused was reported (63% medium/big improvement), as well as an improvement of the mental performance enhancement (58% medium/big improvement). If taken as a last-ditch effort to improve disease symptoms, 10 respondents (63%) rated it as effective. If taken to support a prescribed medicine, 17% reported increased adverse effects from their prescribed medication, 33% reported increased interactions, and 33% reported a stronger effect of their prescribed medication. However, with n = 6, this subpopulation was very small. If taken because of disease symptoms, 68 respondents (79%) perceived efficiency. In this subgroup, improvements were reported across a range of health symptoms (Figure 5B), including general pain (56, 65%), chronic pain (46; 53%), muscle and joint pain (47; 55%), sleep disorders (39; 45%), nervousness (39; 45%) and headaches (39; 45%).

### Perceived CBD efficiency for improving quality of life

#### Limited quality of life due to movement restriction

**FIGURE 6:**
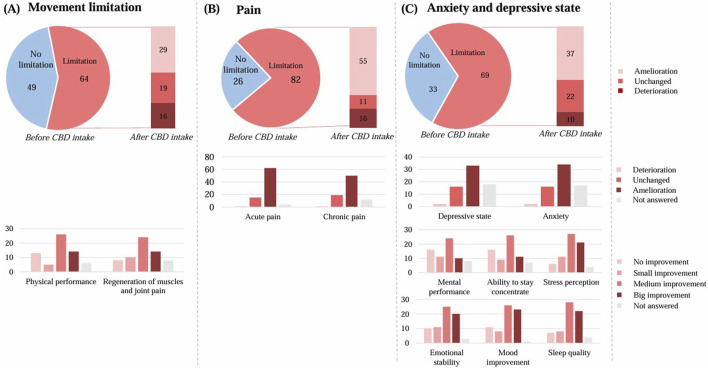
Effect of the CBD product on quality of life. **(A)** regarding movement restriction (n = 113), **(B)** regarding pain (n = 108), **(C)** regarding anxiety and depressive state (n = 102). The graphs show the results in number of respondents.

Out of a total of 113 respondents, 64 of them reported a limited quality of life due to movement restriction. (Figure 6A). Of these, 29 respondents experienced an improvement with CBD use. The physical performance improved upon CBD use by 45 respondents (40%). Regeneration of muscles and joints also improved upon CBD use (42; 37%).

#### Limited quality of life due to self-care restrictions

Out of a total of 108 respondents, 27 of them reported a limited quality of life in terms of self-care restrictions due to difficulties in washing or dressing themselves. Of these, 8 respondents experienced an improvement with CBD use, it remained unchanged by 13 respondents, and 6 respondents experienced a deterioration.

#### Limited quality of life due to restrictions in daily activities

Out of a total of 107 respondents, 57 of them reported a limited quality of life in terms of everyday activities, having difficulties going to work or doing hobbies, or doing things with friends and family. Of these, 28 respondents experienced an improvement with CBD use, it remained unchanged by 18 respondents, and 11 respondents experienced a deterioration.

#### Limited quality of life due to pain

Out of a total of 108 respondents, 82 of them reported a limited quality of life related to pain (Figure 6B). Of these, 55 respondents experienced an improvement with CBD use. By those with a limited quality of life, the acute pain improved by 62 respondents and the chronic pain by 50 respondents.

#### Limited quality of life due to anxiety and depressive state

Out of a total of 102 respondents, 69 respondents of them reported a limited quality of life in terms of anxiety and depressive state (Figure 6C). Of these, 37 respondents experienced an improvement with CBD use. By those with a limited quality of life, the depressive state improved by 33 respondents and the anxiety by 34 respondents.

The mental performance enhancement improved upon CBD use by 45 respondents (44%). The ability to stay concentrate also improved by 46 respondents (45%), as well as the stress perception by 59 respondents (58%).

### Perceived CBD efficiency against disease-related symptoms

**FIGURE 7:**
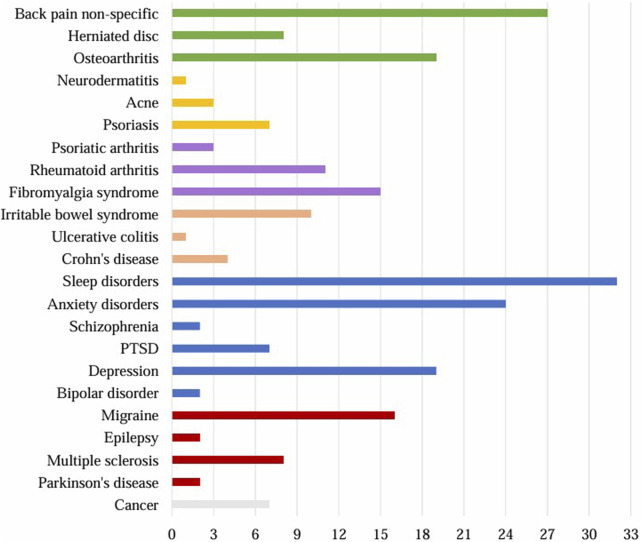
Diagnosed diseases reported by the respondents (n = 230). Grey: cancer, red: neurological diseases, blue: psychiatric diseases, orange: diseases of the gastrointestinal tract, violet: rheumatological diseases, yellow: skin diseases, green: musculoskeletal diseases. PTSD = post-traumatic stress disorder.

A total of 97 respondents (71%) reported having been diagnosed with a disease. A total of 230 diseases (Figure 7) were reported, with an average of more than 2 diseases per person. The most common categories were psychiatric disorders (n = 86), including sleep disorders (n = 32), anxiety disorders (n = 24), and depression (n = 19). The musculoskeletal diseases were also commonly reported (n = 54), including back pain (n = 27), and osteoarthritis (n = 19). Migraine (n = 16), and fibromyalgia syndrome (n = 15) were also common diseases.

Among the 97 subjects with a diagnosed condition, 57% were not taking any medication.

Among the subjects with disease taking medication (n = 41), the CBD product was perceived as effective by 80%, non-effective by 7%. 2% experienced more adverse effects of their prescribed medicine after CBD intake, 2% a weaker effect, and 17% a stronger effect of the prescribed medicine. 68% reported no difference.

Among the 55 subjects taking no medication, the CBD product was perceived as effective by 84% of the cases, and non-effective by 5%.

**FIGURE 8:**
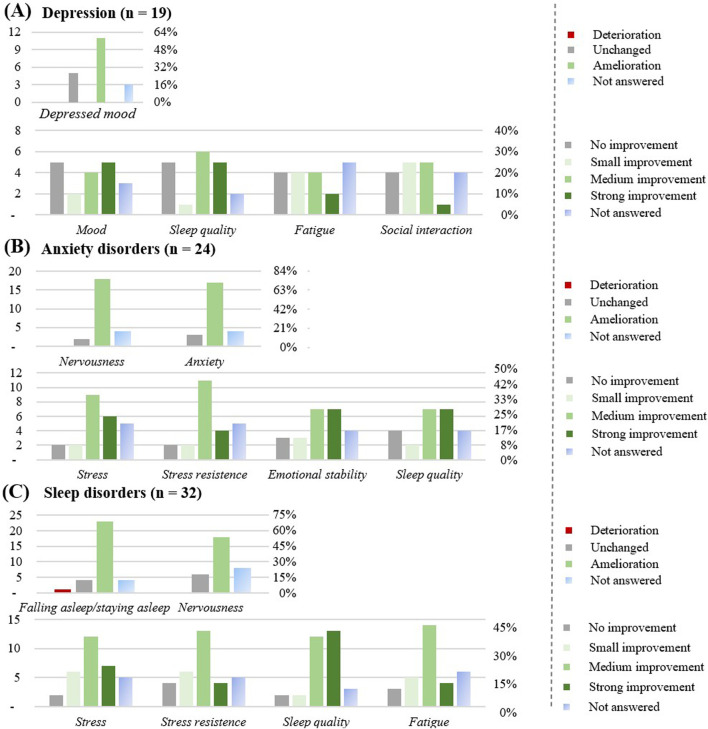
Effect of the CBD products on specific diseases: **(A)** Depression (n = 19). **(B)** Anxiety disorders (n = 24). **(C)** Sleep disorders (n = 32). The graphs show the results in number of respondents (left) and in percentage (right).

**FIGURE 9:**
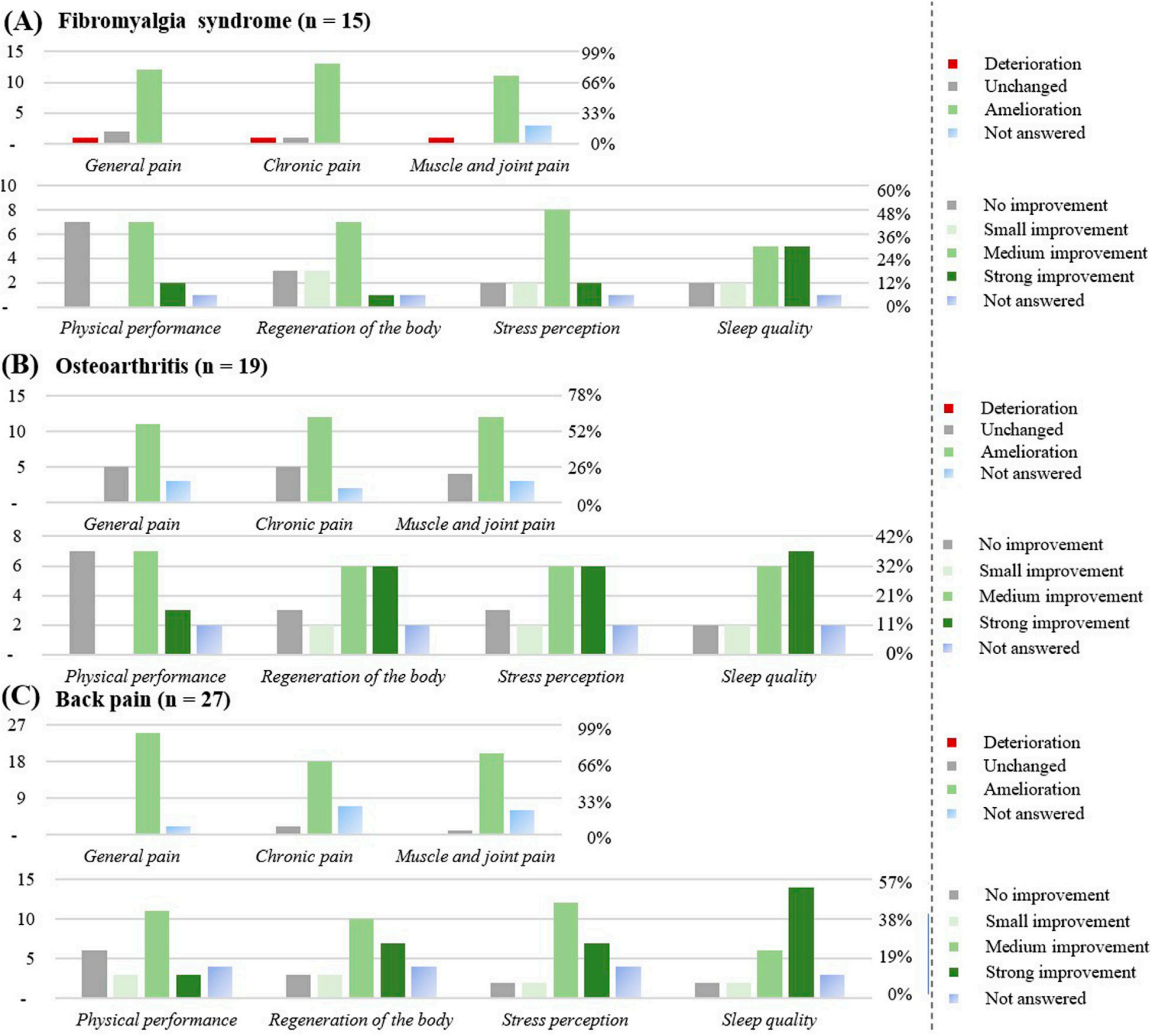
Effect of the CBD products on specific diseases: **(A)** Fibromyalgia syndrome (n = 15). **(B)** Osteoarthritis (n = 19). **(C)** Back pain (n = 27). The graphs show the results in number of respondents (left) and in percentage (right).

In the following chapter, we will focus only on the diseases with n ≥ 15 participants (Figures 8, 9).

#### Respondents with migraine (n = 16)

The favorite form of CBD consumption was oil (n = 15). A total of 12 respondents (75%) reported a chronic use (≥3 months), CBD was often used at least once a day (10; 63%), or as needed (6; 38%). Around 69% of respondents suffered from co-morbidities, most commonly back pain, depression, anxiety disorders, and sleep disorders. Overall, 9 respondents (56%) were on medication, no interaction with the prescribed medicine was noticed. The CBD product (Table 3) was considered effective most of the time (12; 75%). Improvement was observed for headaches (7; 44%) and migraines (10; 63%), mostly after 1 week or directly after the first intake. A medium improvement in the ability to stay concentrated (9; 56%), and in the sleep quality (8; 50%) were noticed.

**TABLE 3:** Effect of the CBD products on respondents with migraine (n = 16).

Effect by respondents with migraine	n (%)
General effect	
Not effective	2 (13)
Effective	6 (38)
Very effective	6 (38)
Not answered	2 (13)
On medication	
Yes	9 (56)
No interaction	8 (89)
Not answered	1 (11)
No	7 (44)
Improvement of headache	
Unchanged	4 (25)
Amelioration	7 (44)
Not answered	5 (31)
Improvement of migraine	
Unchanged	4 (25)
Amelioration	10 (63)
Not answered	2 (13)
Time until improvement	
No change detected	2 (13)
Directly after the first intake	4 (25)
After 1 week	5 (31)
After 1 month	2 (13)
After more than 3 months	1 (6)
Not answered	2 (13)
Ability to stay concentrate	
No improvement	4 (25)
Medium improvement	9 (56)
Not answered	3 (19)
Sleep quality	
No improvement	3 (19)
Small improvement	1 (6)
Medium improvement	8 (50)
Big improvement	1 (6)
Not answered	3 (19)

#### Respondents with depression (n = 19)

The favorite form of CBD consumption was oil (n = 18), followed by flowers (n = 5), with chronic use (≥3 months) reported by 14 respondents (73%). CBD was used at least once a day (12; 64%). 89% of respondents suffered from co-morbidities, most commonly anxiety disorders, sleep disorders, and back pain. A total of 6 respondents (32%) were on medication, no interaction with the prescribed medicine was noticed, except 1 respondent who noticed more adverse effects. The CBD product was considered effective most of the time (14; 74%). Improvement was observed for depressed mood, mostly after 1 week (7; 37%). The mood and the sleep quality improved upon CBD use (11; 58%), as well as fatigue (10; 53%) and social interactions (11; 53%).

#### Respondents with anxiety disorders (n = 24)

The favorite form of CBD consumption was oil (n = 23), followed by flowers (n = 6), with chronic use (≥3 months) reported by 17 respondents (71%). CBD was used at least once a day (16; 67%), or as needed (5; 21%). All respondents suffered from co-morbidities, most commonly sleep disorders and depression. 11 respondents (46%) were on medication, 5 didn’t notice any interaction with the prescribed medicine, 3 noticed a stronger effect of the prescribed medicine, and 1 more adverse effects. The CBD product was most of the time considered to be effective (18; 75%).

Improvement was observed for nervousness (18; 75%) and for anxiety (17; 71%), mostly after 1 month (7; 29%) or 1 week (6; 25%). An improvement in perceived stress (17; 71%), in stress resistance (17; 71%), in emotional stability (17; 71%), and sleep quality (16; 67%) were noticed.

#### Respondents with sleep disorders (n = 32)

The favorite form of CBD consumption was oil (n = 29) followed by flowers (n = 10), with chronic use (≥3 months) reported by 29 respondents (91%). CBD was used at least once a day (18; 57%), or as needed (7; 22%). Around 76% of respondents suffered from co-morbidities, most commonly anxiety disorders, fibromyalgia syndrome and back pain. Overall, 11 respondents were on medication, no interaction with the prescribed medicine was most of the time noticed (n = 8), 2 respondents (6%) noticed a stronger effect of the prescribed medicine. The CBD product was most of the time considered to be effective (27; 85%). Improvement was observed for the difficulty in falling asleep or staying asleep (23; 72%), for nervousness (18; 56%), mostly after the first take (11; 34%), or after a month (9; 28%). An improvement in the perceived stress (25; 78%), in the stress resistance (23; 72%), sleep quality (25; 78%) and fatigue (23; 72%) were noticed by this subgroup.

#### Respondents with irritable or inflammatory bowel diseases (n = 15)

This category includes Crohn’s disease (n = 4), ulcerative colitis (n = 1), and irritable bowel syndrome (n = 10). The favorite form of CBD consumption was oil (n = 14), followed by powder (n = 2), with chronic use (≥3 months) reported by 10 respondents (60%). CBD was used at least once a day (9; 60%). 80% of respondents suffered from co-morbidities, most commonly sleep disorders, migraine, depression, and back pain. A total of 8 participants were on medication, no interaction with the prescribed medicine was noticed. The CBD product (Table 4) was considered to be effective by 13 respondents (87%). Improvement was observed for intolerances (3; 20%), and for digestive problems (9; 60%).

**TABLE 4:** Effect of the CBD products on respondents with irritable or inflammatory bowel diseases (n = 15).

Effect by respondents with irritable or inflammatory bowel diseases	n (%)
General effect	
Not effective	1 (7)
Effective	5 (33)
Very effective	8 (53)
Not answered	1 (7)
On medication	
Yes	8 (53)
No interaction	7 (47)
Not answered	1 (7)
No	6 (40)
Not answered	1 (7)
Improvement of intolerances	
Unchanged	6 (40)
Amelioration	3 (20)
Not answered	6 (40)
Improvement of digestive problems	
Unchanged	3 (20)
Amelioration	9 (60)
Not answered	3 (20)
Time until improvement	
No change detected	1 (7)
Directly after the first intake	5 (33)
After 1 week	3 (20)
After 1 month	4 (47)
Not answered	2 (13)

#### Respondents with fibromyalgia syndrome (n = 15)

The favorite form of CBD consumption was oil (n = 14), with chronic use (≥3 months) reported by 13 respondents (86%). CBD was used at least once a day (9; 60%), or as needed (3; 20%). All respondents suffered from co-morbidities, most commonly sleep disorders or back pain. Overall, 6 respondents (40%) were on medication, 1 respondent (7%) reported a stronger effect of the prescribed medicine. The CBD product was considered to be effective by 13 respondents (86%).

Improvement was observed for general pain (12; 80%), for chronic pain (13; 86%), and for muscle and joint pain (11; 73%), mostly after the first intake (7; 33%). An improvement in physical performance (10; 60%), regeneration of the body (11; 73%), in the stress perception (12; 80%) and in the sleep quality (12; 80%) were noticed by this subgroup.

#### Respondents with osteoarthritis (n = 19)

The favorite form of consumption was oil (n = 16), with chronic use (≥3 months) reported by 14 respondents (74%). CBD was used at least once a day (10; 58%). Around 89% of respondents suffered from co-morbidities, most commonly rheumatoid arthritis and back pain. A total of 8 participants (42%) were on medication, 1 participant noticed a stronger effect of the prescribed medicine. The CBD product was considered to be effective for 14 respondents (74%) (1 respondent; 11% ineffective). Improvement was observed for general pain (11; 58%), for chronic pain (12; 63%), and for muscle and joint pain (12; 63%), mostly after the first take (4; 21%), after a week (4; 21%), or a month (4; 21%).

An improvement in the regeneration of muscle and joints (14; 74%) and sleep quality was noticed (15; 79%) in this subgroup.

#### Respondents with back pain (n = 27)

The favorite form of consumption was oil (n = 26), with chronic CBD use (≥3 months) reported by 23 respondents (85%). CBD was used at least once a day (14; 56%), or as needed (6; 22%). Approximately 89% of respondents suffered from co-morbidities, most commonly fibromyalgia syndrome, rheumatoid arthritis, or osteoarthritis. Overall, 8 respondents (30%) were on medication, 1 participant noticed more adverse effects upon CBD use. The CBD product was most of the time considered to be effective (26; 96%). Improvement was observed for general pain (25; 93%), for chronic pain (18; 71%), and for muscle and joint pain (20; 74%), mostly after the first take (10; 37%), after a week (8; 30%), or a month (5; 19%). An improvement in physical performance (17; 63%), in regeneration of muscle and joints (20; 74%), in sleep quality (22; 81%) was noticed of this subgroup.

### THC/cannabis use

**FIGURE 10:**
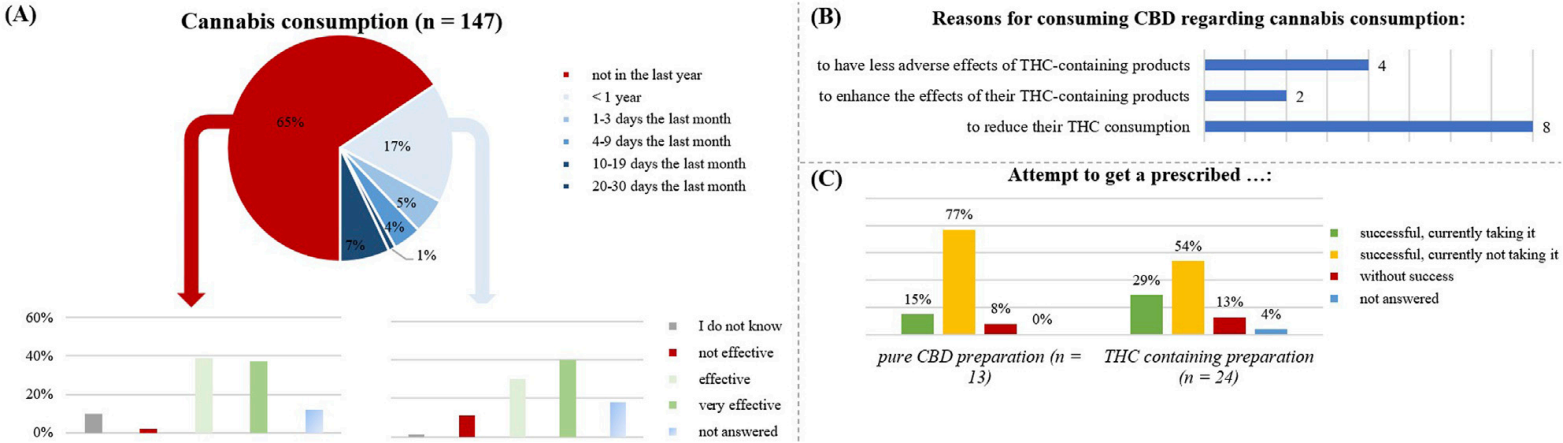
Relation with cannabis by the respondents. **(A)** is focusing on the respondents using the CBD product in relation to a cannabis consumption and analyzing the general effect of CBD without cannabis consumption in the last year (n= 96) and with cannabis consumption in the last year (n = 51). **(B)** shows the number of respondents taking CBD because of a cannabis consumption and their specific reasons. **(C)** is illustrating the attempt to get a prescribed CBD- or THC- containing preparation by the respondents.

A total of 65% of the respondents reported no current cannabis use, 17% had used cannabis for less than 1 year, but more than 1 month, and 17% had used cannabis in the last month (Figure 10A). Among participants who had used cannabis in the past 12 months (n = 51), the CBD product was effective for 76%. Among those who had not used cannabis in the last 12 months (n = 96), the CBD product was effective for 70%.

Around 33% of cannabis users reported having a medical condition. Anxiety disorders were prevalent in 26% of cannabis users, followed by osteoarthritis (21%), depression (28%), fibromyalgia (27%), migraine (19%), back pain (26%), and sleep disorders (29%).

Cannabis use was also sometimes a reason for taking over-the-counter CBD products (Figure 10B): with the goal to reduce their THC consumption (n = 8), to enhance the THC effects by additional CBD use (n = 2), to reduce the adverse effects of THC (n = 4).

Regarding the prescription of CBD/THC medication (Figure 10C), most respondents had not tried to obtain such prescriptions (75%). Among those who had tried, THC-preparations were more common (16%) than pure CBD preparations (9%). Most people who attempted to obtain a prescription were unsuccessful.

In total, 9 subjects received a pharmaceutical preparation containing THC. Of those still using it at the survey date (n = 6), five had multiple diseases including migraine, Crohn’s disease, fibromyalgia syndrome, and back pain. Those no longer taking it (n = 2), also suffered from co-morbidities.

The participants taking a prescribed CBD containing preparation (n = 2), reported 100% effectivity, while a prescribed THC-containing preparation with CBD was effective for 86% (n = 7).

### Perceived adverse effects

**FIGURE 11:**
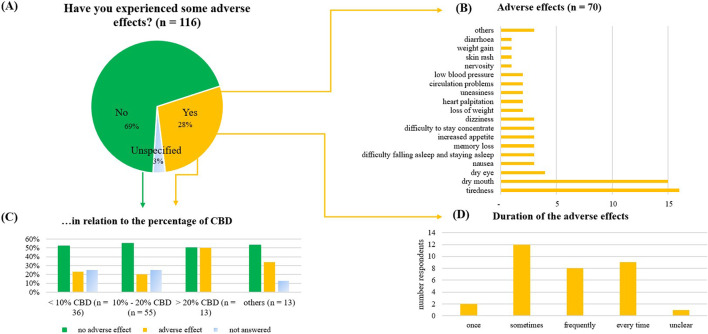
Adverse effects of the consumed CBD product, as reported by the consumers. **(A)** Adverse effect given in percentage (n = 116), **(B)** Further discrimination of the reported adverse effects, **(C)** Reported adverse effects in relation to the percentage of CBD in the product. **(D)** Duration of the reported adverse effects.

In most cases (69%), the respondents reported no adverse effects (Figure 11). Out of these, the most common adverse effects were tiredness (23%), dry mouth (21%), and dry eyes (6%). Tiredness was typically described as mild (38%), or moderate (31%), dry mouth was described as moderate (47%). The adverse effects occurred sometimes (32%), frequently (20%), or every time after consumption (22%). They didn’t improve over time for 47%; for 23%, they didn’t worsen. Adverse effects were reported by 23% of those using products containing less than 10% CBD, 19% of those using products with 10%–20% CBD, and 40% of those using products with more than 20% CBD.

## Discussion

In the present study we report on the results of an exploratory survey on OTC CBD products. Beyond their use for promoting overall wellbeing, CBD products were commonly employed in the treatment of various medical conditions. This is consistent with other studies (Fortin et al., 2021; Moltke and Hindocha, 2021; Corroon and Phillips, 2018). The consumed CBD products were predominantly distributed through general retail channels and online markets. Unlike pharmaceutical CBD preparations, these OTC products are characterized by unclear composition and variability in their cannabinoid content (Liebling et al., 2022; Lindekamp et al., 2024). Moreover, the evidence base for their effectiveness and appropriate use remains limited, as few high-quality, well-controlled studies have been conducted in this area (Engeli et al., 2025). While many studies in cell cultures and pre-clinical animal models have demonstrated a range of promising effects of CBD, including anti-inflammatory and anxiolytic properties (Engeli et al., 2025; Atalay et al., 2019), these findings cannot be directly extrapolated to humans due to differences in pharmacokinetics, dosing, and the complexity of human diseases and behavior (Schwark and Wakshlag, 2023; Iffland and Grotenhermen, 2017). This creates a considerable knowledge gap, leaving both consumers and healthcare professionals with limited resources to guide informed decision-making regarding expected outcomes, dosage, or administration. As a result, it remains uncertain which effects, at what dosages, may arise from CBD itself, or from other bioactive compounds in the product. A meta-analysis of observational clinical studies in treatment-resistant epilepsy reported, for example that CBD-rich extracts were associated with better therapeutic outcomes and fewer adverse effects compared to purified CBD formulations (Pamplona et al., 2018).

A critical and still unresolved question is whether the doses commonly consumed in OTC CBD products are sufficient to produce genuine pharmacological effects, as current evidence does not provide a clear answer. Although our survey did not capture precise data on the amounts of CBD consumed, the study by McGregor et al. (2020) provides an indicative estimate of typical consumption levels among OTC CBD users. This study identified the highest-strength CBD products as 50 mg capsules and oils with 270 mg/mL CBD, resulting in likely daily CBD intakes of less than 150 mg. However, if one considers informal dosage guidelines frequently cited online - based on the classification by Leinow and Birnbaum (2019) - CBD doses between 0.5 mg/day up to 1500 mg/day are recommended, depending on the severity of symptoms (see also [10]). So far, clinical studies suggest that therapeutic benefits of oral CBD become apparent at doses of approximately 300 mg per day (Arnold et al., 2023), although further robust trials are needed to clarify effects at lower doses. Observational data from a cross-sectional study of 387 CBD users reported possible symptom improvements in anxiety and sleep disturbances at daily doses below 100 mg (Moltke and Hindocha, 2021). Similarly, a case series including 47 patients with anxiety and 25 with sleep disorders described improvements at doses as low as 25 mg/day when CBD was administered as adjunctive therapy (Shannon et al., 2019). In an open-label study sublingual treatment of N = 14 patients suffering from moderate-to-severe anxiety with a full-spectrum CBD product significantly improved the symptoms at a dose of around 35 mg/day CBD and 1 mg/day THC, while only minor adverse effects like sleepiness/fatigue, increased energy, and dry mouth were reported (Dahlgren et al., 2022). However, given the absence of placebo controls in these “low-dose effect” studies, the relative contribution of pharmacological effects versus expectation-driven placebo responses remains uncertain.

Placebo effects are well-documented across conditions involving subjective, brain-mediated symptoms such as pain, anxiety, stress, and sleep disturbances—all of which were reasons for CBD use in our study, but also in the studies reported from France (Fortin et al., 2021), United Kingdom (Moltke and Hindocha, 2021), and United States (Corroon and Phillips, 2018). Meta-analyses have consistently demonstrated substantial placebo responses in these domains. For instance, a meta-analysis by Peerdeman et al. (2016) demonstrated robust placebo effects in both acute and chronic pain, largely driven by expectation mechanisms. Likewise, placebo responses in anxiety and depression treatments have been estimated to account for up to 40% of the total treatment effect in clinical trials (Furukawa et al., 2016; Motta et al., 2023). Supporting this, a recent placebo-controlled study demonstrated that expectancy alone—the belief of having ingested CBD—was sufficient to increase sedation and attenuate stress responses during an acute stressor (Zhekova et al., 2024). Furthermore, reports of adverse effects such as dry mouth and tiredness, observed both in our study and in other surveys (Moltke and Hindocha, 2021; Corroon and Phillips, 2018), do not necessarily indicate pharmacological activity, as such symptoms are also commonly reported in placebo groups due to nocebo effects (Häuser et al., 2012; Zhekova et al., 2024). Given the widespread public association between CBD and such adverse effects, it is plausible that consumer expectations shaped both perceived benefits and adverse experiences.

For many endpoints investigated in our survey, about 40% of the participants reported an effect after the first CBD intake. While this could correspond to the known pharmacokinetics of CBD, including its half-life and T_max_ (Millar et al., 2018), it is also plausible that expectancy contributed to the rapid onset of perceived benefits, as placebo responses can manifest quickly, particularly in subjective symptoms such as stress, anxiety, or pain (Benedetti et al., 2011; Benedetti et al., 2022).

Importantly, CBD pharmacology and placebo mechanisms may converge at the neurobiological level. CBD interacts with the endocannabinoid system, which itself is involved in placebo-induced analgesia and stress reduction. Placebo responses in pain modulation have been associated with increased endogenous cannabinoid signaling, including elevated anandamide levels (Benedetti et al., 2011). Since CBD can enhance anandamide availability via fatty-acid amide hydrolase 1 (FAAH) inhibition (Mangiatordi et al., 2023), it is conceivable that both pharmacological effects of CBD and expectation-driven placebo mechanisms converge on the same neurobiological pathways (Pamplona et al., 2018). This overlap could help explain the perceived symptom improvements reported by participants at low CBD doses, where pharmacological activity might be limited, but expectancy effects are pronounced. Gertsch (2018) provides an insightful commentary on this, highlighting that the placebo effect plays a significant role in the perceived efficacy of medical cannabis and cannabinoids, as patient expectations can modulate outcomes via the endocannabinoid system (Gertsch, 2018).

It is important to note that a large majority of the survey participants claimed to be convinced of the health benefits of dietary supplements and expressed a strong belief in the positive effects of CBD. This could indeed indicate that consumer expectations may play a relevant role in the perceived benefits of these products, potentially amplifying placebo responses. The reported belief in the positive effects of CBD could have been a result of perceived positive effects or the belief itself contributed to the perception of effectiveness, again potentially reflecting a placebo response.

Most participants reported taking CBD independently of meals, which suggests that timing of intake has not been a primary consideration for consumers, or that they were unaware of the impact of food intake on CBD absorption. This is surprising since previous pharmacokinetic studies have shown that concomitant food intake, particularly high-fat meals, can significantly enhance systemic CBD exposure (Taylor et al., 2018). In line with this, participants more frequently reported CBD as ineffective when it was consumed more than 2 hours after meals or one to 2 hours before meals. While sublingual administration of CBD is intended to allow absorption through the oral mucosa, recent studies indicate that in practice, much of the CBD may be swallowed before significant mucosal absorption occurs (Johnson et al., 2024). This means that gastrointestinal absorption indeed becomes the predominant route, making factors like food intake relevant to CBD bioavailability. Many consumers used CBD products without professional guidance and relied far more on scientific literature as well as brochures than on consultations with healthcare professionals. In an environment saturated with promotional claims and low-quality studies, it remains uncertain whether lay consumers are able to reliably distinguish between high-quality scientific evidence and less robust information. However, it is also questionable whether healthcare professionals could have provided competent advice on these products, given the variability in product composition and the limited clinical data available for OTC CBD formulations. Future research should investigate the sources consumers consider credible and the criteria they apply to assess scientific validity. This could help to better understand the effect of information (e.g., impact of positive or exaggerated claims about CBD) on perceived CBD effects.

Interestingly, regardless of their level of background knowledge, most participants in our survey expressed no doubt about the safety of the CBD products. This observation concurs with the results from Geppert et al., where survey participants associated CBD consumption with low risk (Geppert et al., 2023). However, this stands in contrast to current scientific and regulatory evaluations, such as the recent review by Engeli et al. (2025), and the joint position paper by the EFSA and United Kingdom authorities (Advisory committee on novel foods and processes and its CBD subcommittee, 2023), both of which highlight substantial uncertainties and potential health risks associated with CBD, particularly concerning liver toxicity and insufficient data on long-term safety. Considering that a high proportion of our survey participants were chronic CBD users and used it at least once a day, this gap between public perception and scientific assessment highlights the importance of evidence-based information strategies to support informed consumer choices.

## Limitations of the study

This study was exploratory, and thus the overall sample size was relatively small. While some subgroups included a relatively large number of participants, allowing for more robust descriptive insights, results from other subgroups with smaller sample sizes should be considered with caution. However, for these cases, the findings may still provide valuable preliminary insights that should be validated in larger studies in the future.

Although we tried to broadly distribute the questionnaire using diverse platforms, and self-help groups for the different diseases, the respondents may not be fully representative of the CBD consumers in the German population (selection bias). It is also possible that some diseases are overrepresented here. In the internet support groups there are usually very committed people who often look for alternative therapies, which may also introduce a bias.

In addition, since this survey was conducted anonymously via the Internet, it is possible that some participants completed the online survey multiple times.

## Conclusion

The results of this exploratory study demonstrate that OTC CBD consumers often associate the CBD use with health improvement or even disease symptom relief. Many of the symptoms targeted by the consumers are highly responsive to expectancy effects and may be substantially influenced by placebo mechanisms. Further research is needed to clarify the effect of the OTC CBD products in a larger and more diverse population. In particular, studies should focus on accurately assessing the ingested doses and their relationship to perceived effects. Moreover, a more detailed examination of diagnosed medical conditions is warranted, potentially including separate analyses for individual disease categories and symptom severity to generate independent and condition-specific insights. Importantly, such studies should incorporate appropriate placebo controls to disentangle genuine pharmacological effects from psychologically mediated responses.

## Data Availability

The original contributions presented in the study are included in the article, further inquiries can be directed to the corresponding authors.
